# Increasing age in men is negatively associated with sperm quality and DNA integrity but not pregnancy outcomes in assisted reproductive technology

**DOI:** 10.3389/fragi.2025.1603916

**Published:** 2025-05-21

**Authors:** Huiyi Xie, Yikai Chen, Shangcheng Xu, Nan Miao, Wenwei Zheng, Chengji Jiang, Tao Sun

**Affiliations:** ^1^ Center for Precision Medicine, School of Medicine, Huaqiao University, Xiamen, China; ^2^ Center for Reproductive Medicine, Quanzhou Women and Children’s Hospital, Quanzhou, China; ^3^ GeneYoung Biopharmaceuticals, Shenzhen, China

**Keywords:** male age, sperm quality, sperm DNA fragmentation index, cumulative pregnancy outcomes, assisted reproductive technology (ART)

## Abstract

As the fertility risks for older males such as infertility and reduced success rates are on the rise, an increasing number of individuals are turning to Assisted Reproductive Technology (ART) to have offspring. However, the influence of paternal age on fertility and pregnancy outcomes in ART cycles remains ambiguous. Here, we analyzed the sperm quality of 6,805 samples and DNA fragmentation index (DFI) of 1,253 samples from Chinese males aged 20–63 years old. Our findings demonstrated that sperm volume, progressive motility, and total motility significantly decline, while sperm DFI increases as paternal age advances. Additionally, by studying 1,205 cases undergoing ART treatment, we discovered that male age and sperm quality do not exhibit a pronounced impact on ART outcomes. Our study has disclosed that sperm quality and DFI are inversely correlated with increasing male age. Our data further suggest that male ages do not significantly affect ART outcomes, which should offer instructive references for ART practice involving older males.

## 1 Introduction

Currently, half of the countries in the world have a fertility rate below the replacement level (Fauser et al., 2024). It is predicted that numerous countries will witness a population decline of over 50% from 2017 to 2,100 (Fauser et al., 2024). This will result in demographic shifts with far-reaching societal consequences. One of the key factors contributing to this scenario is the growing trend of delayed marriage and childbearing. As individuals age, the likelihood of natural conception declines. There are concerns about the fertility risks associated with older men, such as infertility and lower success rates in achieving pregnancy (Ramasamy et al., 2015). Consequently, Assisted Reproductive Technologies (ART), which can effectively facilitate conception, have attracted substantial attention (du Fossé et al., 2020).

The analysis of sperm quality parameters stands as the principal approach for evaluating male fertility (World Health, 2021). Over the past several decades, a discernible and gradual decline in male sperm quality has been witnessed. This decline is characterized by decreases in semen volume, total sperm count, sperm motility, and sperm concentration (Bahri et al., 2021; Liu et al., 2020). Multiple factors can exert an impact on sperm quality, including environmental exposures, lifestyle decisions, genetic elements, and endocrine and metabolic dysfunctions (Chung et al., 2019; Ohlander et al., 2016; Siegel et al., 2022; Zhang et al., 2024).

As paternal age advances, a substantial decline in reproductive cells, encompassing testicular interstitial cells, Sertoli cells, and germ cells, has been observed (Dakouane et al., 2005; Kaler and Neaves, 1978). A decrease in hormone levels, such as total serum testosterone, free testosterone, and androgen concentrations, is apparent (Harman et al., 2001; Kaufman and Vermeulen, 2005). Moreover, it has been discovered that chromosomal aberrations in sperm (Brahem et al., 2011; Ozkosem et al., 2015), DNA fragmentation (Albani et al., 2019; Moskovtsev et al., 2006; Singh et al., 2003), abnormal DNA methylation patterns, the accumulation of deleterious *de novo* mutations, and alterations in sperm telomere length are associated with male aging (Jónsson et al., 2017; Kong et al., 2012; Yang et al., 2015). The primary manifestation of these age-related alterations is the deterioration of semen parameters, such as semen volume and sperm progressive motility (Almeida et al., 2017; Hammiche et al., 2011; Johnson et al., 2015; Verón et al., 2018).

The sperm DNA fragmentation index (DFI) is considered a highly reliable indicator of fertilization capacity and the potential for embryonic development (Aflatoonian et al., 2024; Evenson DP. and Wixon R. 2006). Previous research has demonstrated that when the DFI exceeds 30%, it poses significant challenges to natural conception. This elevated DFI can give rise to pre-implantation embryonic abnormalities and early miscarriage (Esteves et al., 2020; Rex et al., 2017; Zhao et al., 2014). The degree of sperm DNA damage has a substantial impact on the success rates of intrauterine insemination (IUI), *in vitro* fertilization (IVF), and intracytoplasmic sperm injection (ICSI) (Evenson D. and Wixon R. 2006; Zini et al., 2008). Additionally, sperm DNA damage substantially increases the risk of genetic disorders, birth defects, and unfavorable offspring outcomes (Aitken et al., 2014).

Considering the progressive decline in sperm quality with increasing paternal age, the transmission of genetic abnormalities may enhance the likelihood of early pregnancy loss or the occurrence of genetic disorders in offspring (Kaarouch et al., 2015; Lindman et al., 2024). The procedure of ART tends to have females actively involved, such as ovarian stimulation, embryo culture, and embryo transfer, but frequently ignores males, as sperm providers, and simplifies male-related factors and evaluation standards for instance sperm quality controls. As a consequence, limited clinical data are available to assess the male impact on the ART. Therefore, although maternal age is a well-recognized factor influencing the outcomes of the ART, whereas research exploring the relationship between paternal age and pregnancy outcomes remains relatively limited (Ezoe et al., 2023).

In this research, we explored the relationship between male age and sperm quality, along with its potential implications for the success rate in the ART. We identified a marked decline in sperm quality and an elevation in sperm DFI as male age advanced. Notably, neither male age nor sperm quality exerted a conspicuous influence on ART outcomes. Our study has underscored the inverse correlation between male age and sperm quality and has also elucidated the pattern of the ART success rate in relation to increasing male ages.

## 2 Materials and methods

### 2.1 Patients

This retrospective study encompassed male participants who underwent sperm quality and sperm DFI testing, along with couples who received the ART treatment at Quanzhou Women and Children Hospital between 1 January 2018, and 1 July 2022. The study was approved by the Institutional Ethics Committee of the hospital (approval number: 20230304). Written informed consent was obtained from all patients.

### 2.2 Analyses of sperm quality and sperm DFI

In this study, a total of 6,805 sperm quality analyses and 1,253 sperm DFI analyses were conducted. Participants were selected based on having a normal chromosomal karyotype and no history of cryptorchidism, malignancy, radiation exposure, reproductive tract infection, or azoospermia. Patients with bad living habits such as smoking and excessive alcohol consumption, those suffering from diseases such as hypertension and diabetes, as well as those who are obese (with a BMI ≥28) were excluded from this study.

For the sperm quality analysis, the data were divided into five age groups: the 20–24 years old group (n = 102, with an average age of 23.66 ± 0.554 years), the 25–29 years old group (n = 1,789, 27.70 ± 1.212 years), the 30–34 years old group (n = 3,026, 32.83 ± 1.392 years), the 35–39 years old group (n = 1,345, 36.53 ± 1.369 years), and the over-40 years old group (n = 543, 42.22 ± 2.738 years).

For the sperm DFI analysis, the data were also categorized into five age groups: the 20–24 years old group (n = 25, 23.48 ± 0.586 years), the 25–29 years old group (n = 305, 27.77 ± 1.219 years), the 30–34 years old group (n = 557, 31.86 ± 1.402 years), the 35–39 years old group (n = 272, 36.46 ± 1.333 years), and the over-40 years old group (n = 94, 42.67 ± 3.462 years).

The parameters utilized to evaluate sperm quality encompassed semen volume, concentration, progressive motility rate, and total motility rate.

### 2.3 Analysis of ART outcomes

A total of 1,205 treatment cycles were incorporated into this study. The female partners selected in this study were younger than 37 years old, had a normal chromosomal karyotype and normal ovarian reserve, and were undergoing their first infertility treatment. Females with factors that affect female fertility such as endometriosis, diabetes, hypercortisolism, and factors related to serious diseases that could affect continuous pregnancy were excluded in this study.

The data were partitioned into five groups based on the male ages. These groups were as follows: the 20–24 years old group (n = 25, with an average male age of 23.48 ± 0.586 years), the 25–29 years old group (n = 296, 27.77 ± 1.23 years), the 30–34 years old group (n = 533, 31.86 ± 1.40 years), the 35–39 years old group (n = 265, 36.46 ± 1.32 years), and the over-40 years old group (n = 86, 42.70 ± 3.58 years).

The primary outcome measures consisted of embryonic development, cumulative pregnancy outcomes, and neonatal birth weight.

### 2.4 Statistical analysis

All data were analyzed using SPSS version 26.0. Analysis of Variance (ANOVA) was utilized to evaluate differences among various groups, while *Chi*-square tests were applied to conduct statistical analyses on categorical data. The influence of male ages on pregnancy outcomes was explored through a multivariate logistic regression analysis, with male age being treated as a categorical variable. Statistical significance was set at *P* < 0.05.

## 3 Result

### 3.1 Sperm quality significantly declined with increasing age

Given that the decline in male fertility is closely linked to sperm quality parameters such as sperm volume, progressive motility rate, and total motility rate, the analysis of sperm quality represents a crucial indicator in the evaluation of male fertility (Bahri et al., 2021; Liu, Dai, Li, Yuan, Wang, Wang and Guan, 2020). In this study, we first examined the sperm quality of 6,805 Chinese males aged 20–63 years (with a mean age of 32.38 ± 4.52) during the study period using a computer-aided sperm analysis system.

**FIGURE 1:**
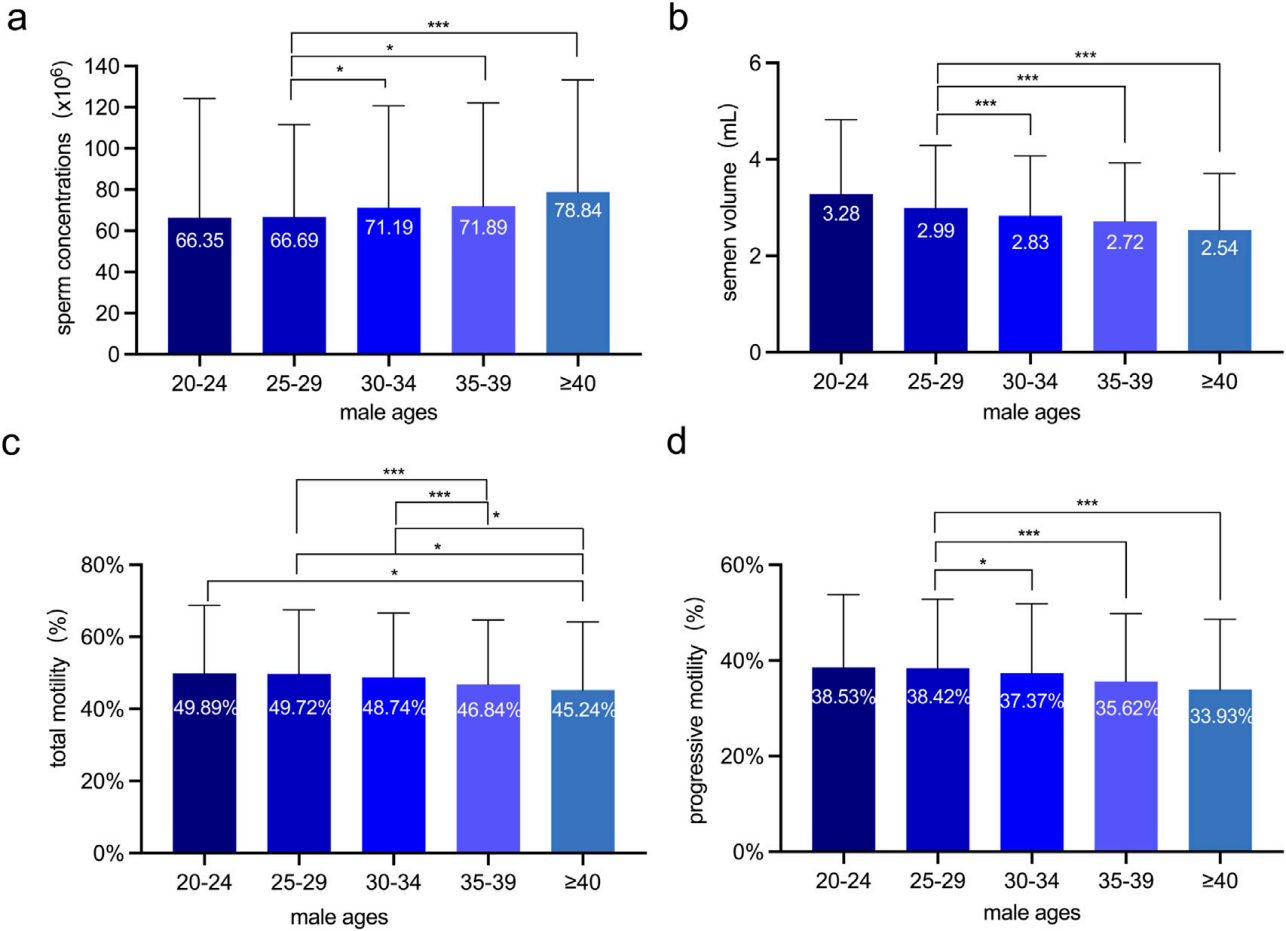
The Sperm quality analyses of 6,805 Chinese males at various ages. **(a)** Sperm concentration (×10^6^/mL) across age groups. **(b)** Semen volume (mL) across age groups. **(c)** Total sperm motility (%) across age groups. **(d)** Progressive sperm motility (%) across age groups. The semen volume, progressive motility and total motility were significantly inversely associated with advancing age. One-way ANOVA was employed to assess variations among different male groups, independent sample t-tests were performed to assess the differences between every two groups. *: p < 0.05 and ***: p < 0.001.

Sperm concentrations were comparable among the 30-34, 35-39, and over-40 age groups. However, they were notably lower in the 25-29 age group (Figure 1a). Semen volumes were similar in the 30-34, 35-39, and over-40 age groups, while being higher in the 25-29 age group (Figure 1b). Total sperm motility demonstrated a significant decrease in the 35-39 and over-40 age groups when compared to the 20-24, 25-29, and 30-34 age groups (Figure 1c). Progressive motility likewise showed an age-related decline, with the 25-29 age group having higher rates than the 30-34, 35-39, and over-40 age groups (Figure 1d).

These results indicate that semen volume, total sperm motility, and progressive motility are inversely correlated with male ages. Specifically, they show a significant decline as male age increases.

### 3.2 Sperm DFI significantly increased with increasing age

**FIGURE 2:**
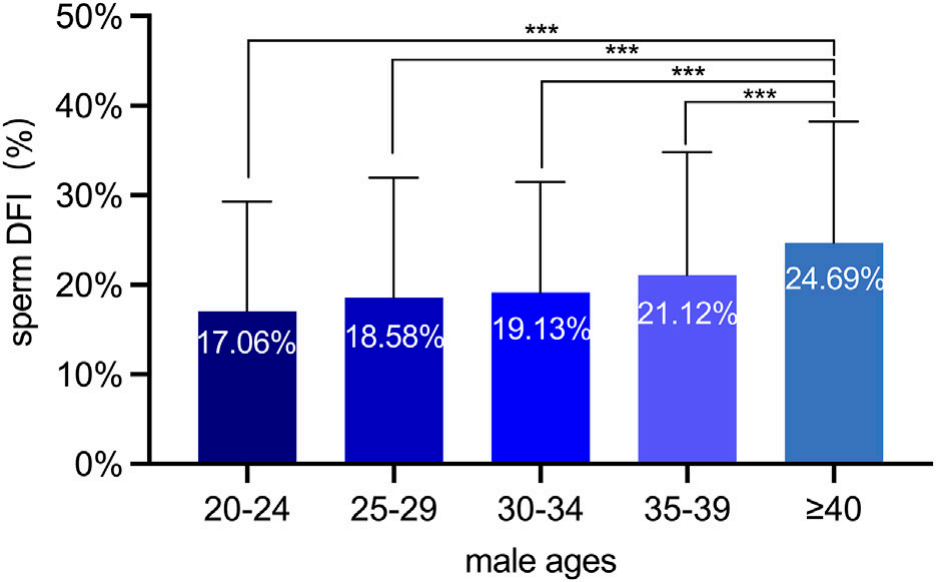
The sperm DFI analyses of 1,253 Chinese males at various ages. Males aged over 40 exhibited a marked increase in DFI compared to the other four age groups, suggesting a progressive deterioration of sperm DNA integrity with advancing age. ***: *p* < 0.001.

Previous studies have identified the sperm DFI as a critical parameter for evaluating the degree of sperm DNA damage. A higher DFI level is associated with lower sperm DNA integrity (Aflatoonian, Amjadi, Sheibak, Moradi, Aflatoonian, Tabatabaei, Berjis, Aflatoonian and Zandieh, 2024; Evenson DP. and Wixon R. 2006). In this study, we employed the sperm chromatin diffusion (SCD) assay to analyze the sperm DFI in 1,253 Chinese males of diverse age groups. Although the sperm DFI did not exhibit significant variations among the 20-24, 25-29, 30-34, and 35-39 age groups, it was substantially elevated in the over-40 age group. This finding implies a discernible deterioration in the integrity of sperm DNA among males aged over 40 years (Figure 2).

### 3.3 Male ages did not affect the success rate in the ART

**FIGURE 3:**
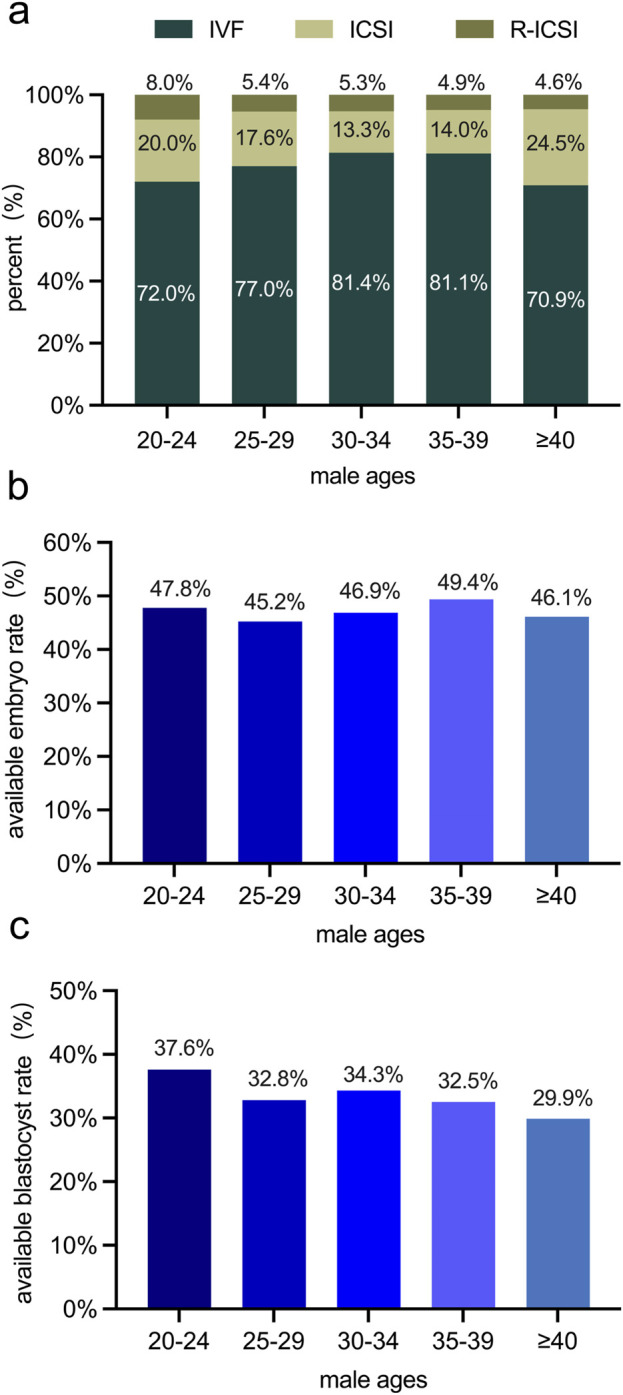
Examinations of variations in embryos among male age groups in 1,205 couples undergoing the ART. **(a)** the percentage of distribution of different fertilization methods (IVF, ICSI, R-ICSI) among different male age groups. **(b)** The available embryo rate (%) among different male age groups. **(c)** The available blastocyst rate (%) among different male age groups. No statistically significant differences were observed in the fertilization method, available embryo rate, or available blastocyst rate among various male age groups.

Studies have demonstrated that fertilization methods, the available embryo rate, and the available blastocyst rate are pivotal indicators of the success rate in the ART (The Istanbul consensus workshop on embryo assessment: proceedings of an expert meeting, 2024). Given our finding of a decline in sperm quality with increasing male age, especially in the over-40 age group, we investigated whether these ART indicators are associated with male age. The fertilization methods encompassed in this study were *in vitro* fertilization (IVF), Intracytoplasmic Sperm Injection (ICSI), and Rescue Intracytoplasmic Sperm Injection (R-ICSI). Intriguingly, among the 1,205 couples under study, the fertilization methods, available embryo rate, and available blastocyst rate did not display significant differences across different male age groups (Figure 3). These results imply that although male age impacts sperm quality, it does not exert a negative influence on the developmental quality of embryos during the *in-vitro* culture phase in the ART.

**FIGURE 4:**
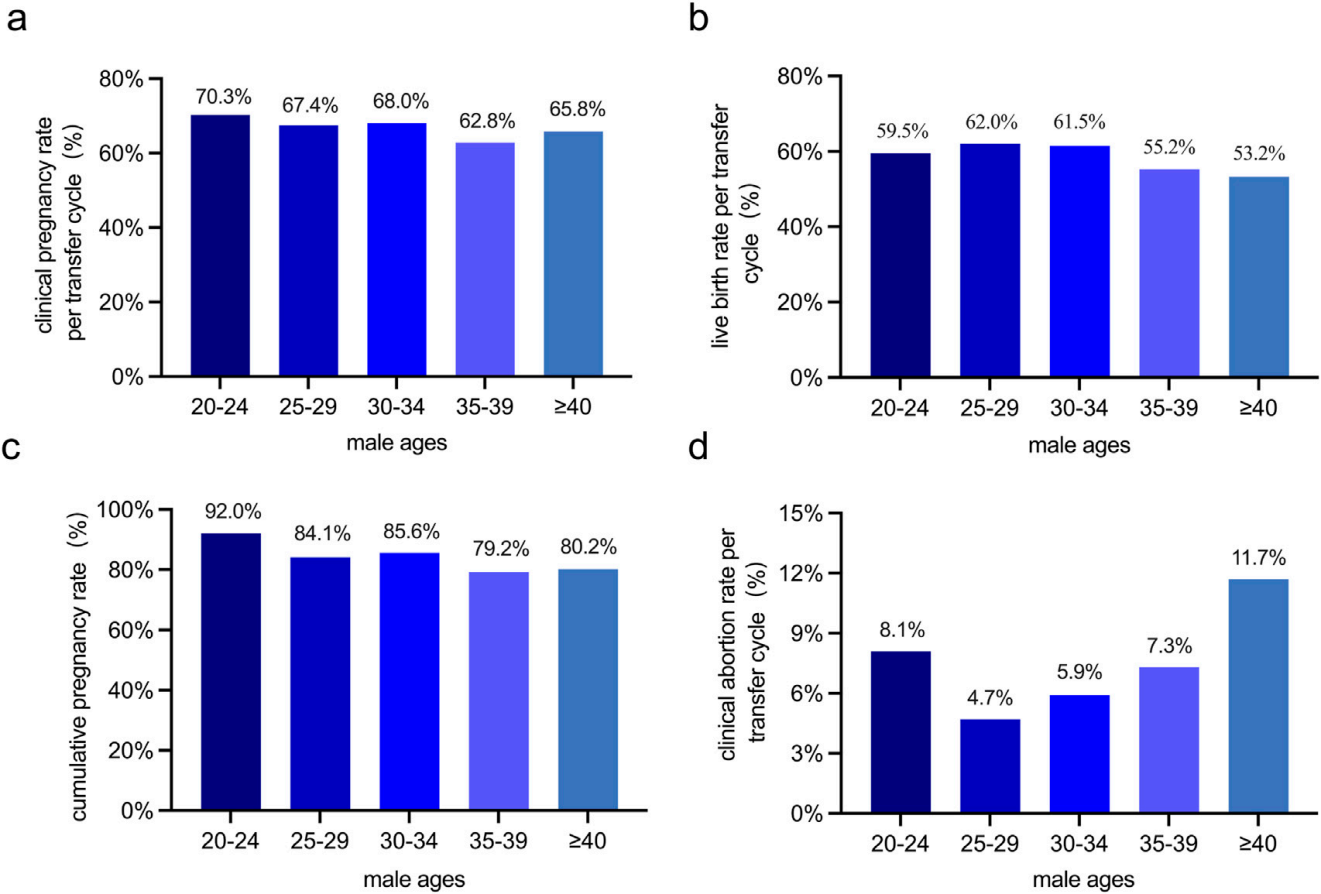
Comparison of pregnancy outcomes across male age groups in 1,205 ART cycles. **(a)** The clinical pregnancy rate per transfer cycle (%) among different male age groups. **(b)** The live birth rate per transfer cycle (%) among different male age groups. **(c)** The cumulative pregnancy rate (%) among different male age groups. **(d)** The clinical abortion rate per transfer cycle (%) among different male age groups. No statistically significant differences in clinical pregnancy rate per transfer cycle, live birth rate, cumulative pregnancy rate, or clinical abortion rate per transfer cycle were detected among different male age groups.

Furthermore, the clinical pregnancy rate per transfer cycle, live birth rate per transfer cycle, and cumulative pregnancy rate are crucial indicators for evaluating the success of the ART (The Istanbul consensus workshop on embryo assessment: proceedings of an expert meeting, 2024). Our findings revealed that as male age increased, the clinical abortion rate per transfer cycle rise, while the clinical pregnancy rate per transfer cycle, live birth rate per transfer cycle, and cumulative pregnancy rate all decrease. However, despite these upward and downward trends in the rates, the observed changes were not statistically significant (Figure 4).

The normal fertilization rate increased in tandem with male age. Specifically, the over-40 age group exhibited significantly higher rates compared to the 20-24, 25-29, and 30-34 age groups. Conversely, the cumulative birth rate decreased as male age advanced. Notably, the rate in the 30-34 age group was significantly higher than that in the over-40 age group (Supplementary Table S1).

Furthermore, we performed statistical analyses on the birth status of the offspring. There were no statistically significant differences in the birth weight and the incidence of abnormal birth weight among single or twin fetuses across different male age groups. This suggests that once ART is successful, male age does not have an impact on the birth weights of the offspring (Supplementary Figure S1).

## 4 Discussion

The challenges faced by older couples in achieving natural conception have escalated owing to age-related deteriorations in physical functions. The ART has emerged as a highly effective solution. Despite the growing prevalence of ART, research regarding the relationship between paternal age and pregnancy outcomes remains relatively underdeveloped. In this study, we analyzed the sperm quality of 6,805 Chinese males ranging in age from 20 to 63 years old. Additionally, we systematically collected data from 1,205 cases undergoing ART treatment. In clinic practice in China, men who visit the infertility clinic are required to take sperm quality tests. However, only men who are of advanced age or have experienced repeated implantation failures are required to take the sperm DFI test. Moreover, some infertile patients choose to receive clinical treatments such as ovulation monitoring, antioxidant therapy, and traditional Chinese medicine treatment. Therefore, a limited number of patients receive the ART in the end.

Our findings indicated a significant decline in sperm quality and an increase in sperm DFI with advancing male age. However, male age and sperm quality did not have a discernible impact on ART outcomes. This study uncovered the correlations among male ages, sperm quality, and ART outcomes. The insights gleaned from this research are expected to offer valuable and instructive information for future ART applications involving older males, potentially guiding medical practitioners in optimizing treatment strategies.

Our comprehensive examinations of semen volume, total motility, and progressive motility revealed that male age exerts a negative influence on sperm quality. Specifically, we found that sperm total motility and progressive motility reach their peak levels prior to the age of 30. Subsequently, a decline commenced after the age of 35, with the most pronounced decrease being observed in men over 40. This phenomenon is likely linked to alterations within the hypothalamic-pituitary-gonadal axis. A reduction in male hormone secretion from the adrenal cortex can potentially impact testicular spermatogenesis, as proposed in reference (AbdulHussain et al., 2020). In addition, we found that the sperm concentration increases with age. This may be due to the reduction in seminal plasma secretion caused by increasing age and reproductive tract infections, resulting in a decrease in semen volume and an increase in sperm concentration (Zitzmann, 2013).

Furthermore, the process of aging induces substantial changes in reproductive organs, including the testicles, vas deferens, and prostate. These anatomical and physiological alterations may further compromise sperm quality (Ashapkin et al., 2023). Additionally, our analysis of DFI demonstrated a significant positive correlation with advancing age, clearly indicating a decline in sperm DNA integrity. Older males are more prone to exposure to environmental hazards and reproductive diseases. Such exposures can modify enzymes like glutathione peroxidase and phospholipase. As a consequence, these changes may ultimately result in increased sperm DNA damage associated with the aging process (Aitken, Smith, Jobling, Baker and De Iuliis, 2014). As age progresses, the antioxidant capacity of the epididymis and the sperm DNA repair mechanisms gradually deteriorate. This continuous decline may further contribute to the elevation of the sperm DFI(Zhou et al., 2024). In conclusion, our findings strongly support an inverse relationship between male age on one hand, and sperm quality and DNA integrity on the other.

In an effort to explore the relationship between male age and the ART outcomes, we deliberately selected female participants who were 37 years old or younger and had generally normal ovarian reserve function. This selection criterion was implemented to minimize the confounding influence of female factors on the study results. During our analysis, we did not observe any significant differences in fertilization methods or the available embryo rate among different male age groups. It is known that early embryo development is predominantly regulated by the maternal genome. Paternal genes typically become activated and expressed only when the embryo reaches the six to eight cell stage. This biological phenomenon implies that male age is unlikely to have a substantial impact on early embryo development within the context of ART. However, our data on the available blastocyst rate suggested a decreasing trend in groups with older male partners.

Previous studies have demonstrated that sperm of low quality can impede blastocyst formation and increase the incidence of sex chromosome aneuploidy. These effects, in turn, raise the probability of adverse pregnancy outcomes (Coates et al., 2015). In our analysis of data from ART procedures, we did not identify any statistically significant differences in the pregnancy rate, miscarriage rate, live-birth rate, and cumulative pregnancy rate across different male age groups for each transplantation cycle. This finding aligns with previous reports (Gallardo et al., 1996; Paulson et al., 2001). However, a statistically significant difference was noted in the cumulative live birth rate. In addition, the oldest males recruited in this studies were 63. A sperm damage threshold might exist in older males, which exceeding this threshold might have an impact on the ART. The future study should recruit more males including those older than 65.

Early embryonic development is likely to be predominantly influenced by maternal genetic material. This is supported by research indicating that the oocyte has the capacity to repair DNA damage inflicted by sperm (Lord and Aitken, 2015). The oocyte’s repair mechanisms may buffer the potential negative impacts of sperm-related factors, such as those associated with different male age groups, on early embryo viability and subsequent live-birth outcomes. As a result, the observed lack of significant differences in many pregnancy-related metrics across male age groups could be attributed to the dominant role of the maternal contribution during the early stages of embryo development.

In conclusion, our research has shown that as male age advances, both sperm quality and DNA integrity deteriorate. Despite the fact that we did not detect any significant disparities in the outcomes of the ART among different male age groups, it is evident that advanced paternal age is linked to a certain level of declined fertility. Our study has revealed the correlations among male ages, sperm quality and ART outcomes, which will provide instructive information for future implementation of ART procedures involving older males.

## Data Availability

The raw data supporting the conclusions of this article will be made available by the authors, without undue reservation.
