# Supplementary material for: Increasing age in men is negatively associated with sperm quality and DNA integrity but not pregnancy outcomes in assisted reproductive technology
Source: Front Aging. 2025 May 21;6:1603916. doi: 10.3389/fragi.2025.1603916 (PMC12133931; doi:10.3389/fragi.2025.1603916)
Supplement: Supplementary file 1 [file Table1.docx]

Table S1 Comparison of normal fertilization rate and cumulative live birth rate among different age groups

| **age** | **20-24** | | **25-29** | | **30-34** | | **35-39** | | **≥40** | | **F/χ2** | | **P** | |  |
| --- | --- | --- | --- | --- | --- | --- | --- | --- | --- | --- | --- | --- | --- | --- | --- |
| n | 25 | | 296 | | 533 | | 265 | | 86 | |  | |  | |  |
| normal fertilization rate | | 56.9^e^ | | 59.2^e^ | | 61.0^e^ | | 62.3 | | 67.0^abc^ | | 24.999 | | <0.001* | |
| cumulative live birth rate | | 88.0 | | 81.1 | | 81.8^e^ | | 74.0 | | 68.6^c^ | | 14.208 | | 0.007* | |
