# Supplementary figures and images for: Increasing age in men is negatively associated with sperm quality and DNA integrity but not pregnancy outcomes in assisted reproductive technology
Source: Front Aging. 2025 May 21;6:1603916. doi: 10.3389/fragi.2025.1603916 (PMC12133931; doi:10.3389/fragi.2025.1603916)

**a****Single fetus**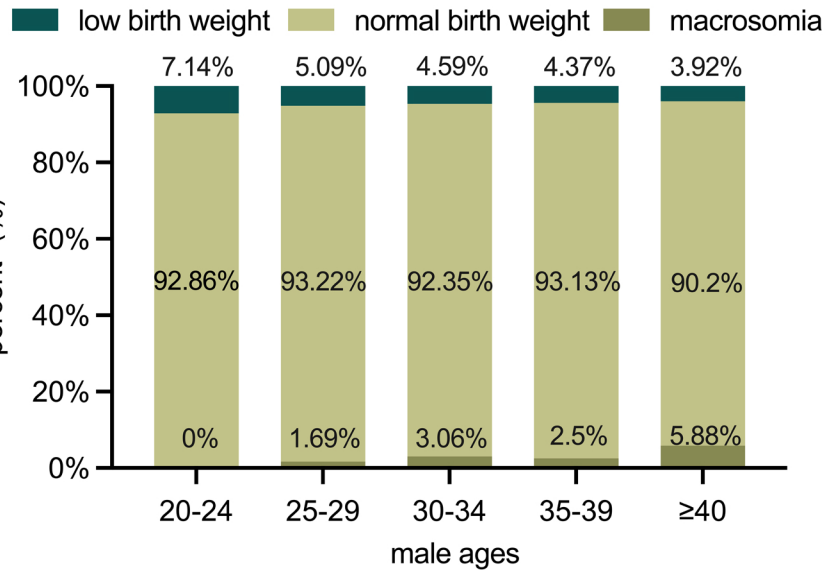**b****Twin fetuses**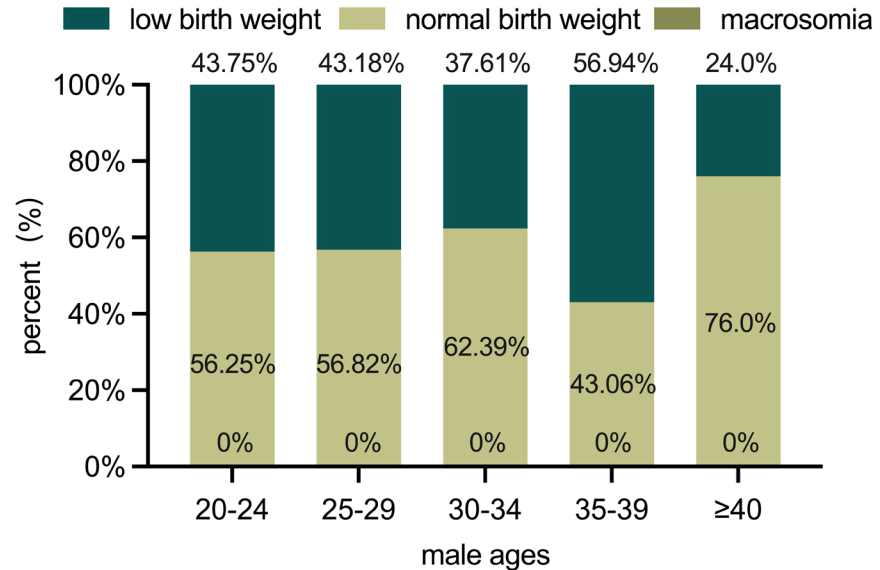

Supplement: Supplementary file 2 [file DataSheet1.pdf]
